# Determinants of body mass index by gender in the Dikgale Health and Demographic Surveillance System site, South Africa

**DOI:** 10.1080/16549716.2018.1537613

**Published:** 2018-11-05

**Authors:** Felistas Mashinya, Marianne Alberts, Ian Cook, Sam Ntuli

**Affiliations:** a Faculty of Health Sciences, University of Limpopo, Polokwane, South Africa; b Faculty of Humanities, University of Limpopo, Polokwane, South Africa

**Keywords:** BMI distribution across African communities, Body mass index, hierarchical modelling, rural, older adults

## Abstract

**Background**: The study was conducted in the Dikgale Health and Demographic Surveillance System (DHDSS) site where we have observed increasing obesity levels, particularly in women, despite evidence of high physical activity (PA) and a relatively low daily energy intake.

**Objective**: This study aimed to assess the socio-demographic, behavioural and biological determinants of body mass index (BMI) in adult residents permanently residing in the DHDSS.

**Methods**: A cross-sectional study was conducted in which socio-demographic, behavioural and biological characteristics from 1143 participants (aged 40–60 years) were collected using a paper questionnaire and standard anthropometric measures. Human immunodeficiency virus (HIV) testing was performed on all participants except those who indicated that they had tested positive. Chi-square and Mann-Whitney tests were used to analyze categorical and continuous variables, respectively, while hierarchical multivariate regression was used to analyze predictors of BMI.

**Results**: The median age of women and men was 51 (46–56) and 50 (45–55) years, respectively. The prevalence of overweight-obesity was 76% in women and 21% in men. A significant negative association of BMI with HIV and smoking and a significant positive association with socio-economic status (SES) was observed in both sexes. In women, BMI was negatively associated with sleep duration (p = 0.015) and age (p = 0.012), but positively associated with sugar-sweetened beverages (SSBs) (p = 0.08). In men, BMI was negatively associated with alcohol use (p = 0.016) and positively associated with being married (p < 0.001). PA was not associated with BMI in either sexes. Full models explained 9.2% and 20% of the variance in BMI in women and men, respectively.

**Conclusion**: BMI in DHDSS adults is not associated with physical inactivity but is associated wealth, marital status, sleep, smoking, alcohol use, and HIV status. Future studies should explore the contribution of nutrition, stunting, psycho-social and genetic factors to overweight and obesity in DHDSS.

## Background

Obesity remains a public health challenge, because of its association with non-communicable diseases (NCDs), such as hypertension, diabetes mellitus and cardiovascular diseases, that are amongst the leading causes of death worldwide [1–4]. Globally, over 600 million adults are obese, with rapidly increasing levels of obesity occurring in Low-to-Middle Income Countries (LMIC) [5,6]. In South Africa, the prevalence of overweight and obesity is 65.1% and 31.3%, for females and males, respectively [7]. The high female-to-male ratio of obesity, is often observed in LMIC [8], but in high-income countries, there is seldom a difference between men and women, or men are more likely to be obese [9]. These regional differences could result from variations in risk factors of obesity such as alcohol use, physical activity, diets and sociocultural perceptions [8,10].

Literature suggests that obesity is associated with multiple factors such as diet, physical inactivity (PI), stress, lack of sleep, cultural beliefs, childhood stunting, genetics factors and the microbiome [8,11,12]. However, some suggest that the Physical Inactivity and Nutritional Transitions are the major causes of obesity [8,13]. Moreover, some cross-sectional and longitudinal studies question the aetiological role of PI, while the role of specific macro-nutrients in the development of obesity remains controversial [14–16]. In addition, factors such as sedentarism, stress, lack of sleep and a cultural belief that bigger-is-healthier have been associated with increased body mass index (BMI) [8,11,17]. An independent, positive relationship between socioeconomic status (SES) and BMI was reported in rural but not urban women, suggesting variations between rural and urban areas [16]. Stunting, which is high in African countries, is a significant contributor to obesity in adolescence and adulthood [12]. The role of emerging risk factors such as genetics and the microbiome are based on the hypothesis that their interactions with the environment and dietary changes increase the risk for obesity, but the pathophysiology for this complex interaction remains unclear [18].

In the DHDSS site, levels of overweight and obesity are high particularly in females (54%) [19], as is stunting (48%) in children [20]. Earlier studies from the DHDSS site were fragmented and considered only diet or physical activity (PA) [15,21,22], while this study considers the relationship of socio-demographic, lifestyle and biological factors with BMI through a sex-specific, hierarchical multiple linear regression modelling approach. Consequently, this study provides baseline DHDSS site cohort data for Phase 2 of the Africa Wits-INDEPTH (International Network for the Demographic Evaluation of Populations and Their Health) partnership for Genomic (AWI-Gen) Study.

## Methods

### Study design and site

A population-based, cross-sectional study was conducted between November 2014 and August 2016. The study was conducted in the DHDSS site [23], one of the collaborative sites under Africa Wits-INDEPTH partnership for Genomic (AWI-Gen) Study, and focuses on the interplay between genomic and environmental risk factors for cardiometabolic diseases among 40–60 year-old in four sub-Saharan African countries [24]. Currently, the DHDSS site covers 15 villages with an estimated 40000 people predominantly Ba-Pedi ethnicity who speak Se-Pedi [23]. The DHDSS site is situated 40km north-east of Polokwane, the capital city of the Limpopo Province, South Africa.

### Participant inclusion and exclusion criteria

The study included DHDSS permanent residents aged 40–60 years. A permanent resident was defined according to HDSS criteria, as an individual residing continuously in a household for at least 6 months of the year. Pregnant women and individuals presenting with physical impairments that would prevent the measurement of anthropometric indices were excluded from the study.

### Study participants

A total of 5479 individuals aged 40–60 years were identified from the DHDSS site database. As the community is known to be highly mobile [23], trained field workers visited households that included 40–60 year-old permanent residents and invited the qualifying individuals to participate in the study. On the scheduled date, participants were transported to the study centre at the University of Limpopo, where the researchers explained the information contained in the information and consent forms and requested the participants to complete the consent form. A total of 1398 individuals participated, however 29 were excluded due to incomplete data sets. Of the remaining 1369, 226 (156 women and 70 men) were excluded for age < 40 years or > 60 years. A complete dataset was available for 1143 participants aged 40–60 years.

### Data collection

An AWI-Gen paper questionnaire consisting of questions from the WHO Steps questionnaire and additional study-specific questions, was developed [25]. Data on socio-demographic-, behavioural- and biological factors were collected via the translated (Se-Pedi) and pilot-tested paper questionnaire by trained field workers, and captured into REDCap, a secure web-based application [24]. Approximately 10% of all data entries were checked for accuracy as described in detail elsewhere [26].

### Sociodemographic factors

Marital status was categorized as never married or cohabited; married or living with partner; divorced or widowed. The level of education was recorded under four categories as no formal education; primary; secondary; or tertiary education. Employment status was categorized as either unemployed or employed (either formal or informal employment or self-employed). SES was calculated as according to the INDEPTH Demographic and Health Surveys program that involves a principal component analysis of the household assets, predicting factor scores and then categorizing these scores into quintiles [27]. Housing density was considered as the number of people in a household divided by number of rooms.

### Behavioural factors

Alcohol consumption was categorized as never consumed; current non-problematic, current problematic or former consumer. Smoking status has three categories as never; current or former smokers. Bread intake was reported as servings/day and calculated by dividing the product of the number of days bread is eaten and the number of servings, by 7. Current guidelines suggest 10 servings of carbohydrate rich foods/day (1 slice of bread = 1 serving) [28]. Fruit and vegetable intake in servings per day was calculated as follows: (Number of days eaten fruit x number of servings/7) + (Number of days eaten vegetables x number of servings/7). Less than five servings/day is considered low intake [25]. Sugary sweetened beverages refer to combined sugary drinks and fruit juices calculated by summing sugary drinks intake per day and fruit juice intake per day. Current guidelines suggest 1 serving (355mL)/day [29]. Self-reported moderate to vigorous physical activity (MVPA) was assessed using the Global Physical Activity Questionnaire (GPAQ), a WHO validated tool for use in developing countries [30]. Total MVPA in minutes per week was a summation of MVPA for transport, work and leisure time, according to the WHO STEPS manual [25]. MVPA was categorized as physically inactive (< 150 minutes/week) or active (≥ 150 minutes/week). The hours of weekday and weekend sleep were calculated from the time individuals go to sleep and wake-up during the week and weekends, respectively. Average sleep in hours per night was calculated as follows: (Hours of weekday sleep × 5 + hours of weekend sleep × 2)/7. The recommended sleeping duration for adults aged 40–60 years is 7–9 hours/night [31].

### Biological factors

Menopausal status was determined using the last date of menstruation. Menopausal status was categorized as pre-menopausal for women with regular menstrual periods; peri-menopausal for women with irregular periods within the past year; post-menopausal for women with no periods within the past year. Number of pregnancies was recorded for both live and still births. HIV testing was performed on all participants except those who indicated that they had tested positive. Participants undergoing HIV testing received pre-counselling prior to blood collection. Blood for HIV testing was collected in EDTA tubes. Samples were initially tested for HIV using the Determine HIV1/2 Ag/Ab Combo kit. Positive samples were re-tested using the Double Check Gold Ultra HIV 1/2 kit. Both kits are ELISA-based and supplied by Inverness Medical, Tokyo, Japan. Post-test counselling was provided and HIV-positive individuals were referred to the local Primary Health Clinic.

### Anthropometric measurements

Weight was measured to the nearest 0.1kg using an Omron BF 400 scale (Omron Healthcare). Participants were requested to remove their shoes and heavy clothing. Height was measured to the nearest 0.1cm using a stadiometer. From weight (kg) and height (m^2^), BMI was calculated by dividing weight (kg) by height (m^2^). BMI was categorized as follows: Underweight < 19 kg/m^2^, Normal weight: 19–24.9kg/m^2^, Overweight: 25–29.9kg/m2 and Obese: ≥ 30kg/m^2^ [32].

### Data analysis

All statistical analyses were performed using Stata software, version 7.0 (Stata Corp., College Station, USA). All the continuous variables were assessed for normality using probability plots and the Shapiro-Francia *W’* test. Non-normally distributed continuous variables were presented as median and interquartile range (IQR). To compare proportions and medians between sexes, Chi-square tests and Mann-Whitney tests were used, respectively. Because BMI was not normally distributed, log transformation was performed for both sexes and used in subsequent analyses. Sex-specific, bivariate linear regression analyses were used to assess the associations between BMI, socio-demographic-, behavioural- and biological factors. Subsequently, factors with a p-value of < 0.2 were included in the hierarchical modelling. Age was added to the models because of its clinical significance, irrespective of the associated bi-variate p-value. To estimate the independent effects of factors on BMI, a Sex-specific, hierarchical linear regression analysis was [33]. Model 1 was built from sociodemographic factors including age, marital status, level of education, SES, and employment status (only in male model). Model 2 consisted of sociodemographic factors and behavioural factors including bread intake, fruit and vegetable intake (only in female model), sugar sweetened beverages, smoking status alcohol use, and sleep. Model 3 consisted of sociodemographic, behavioural and biological factors including HIV, and number of pregnancies (only in female model). Multicollinearity was assessed using the Variance Inflation Factor (VIF) [34]. In general, a VIF ≥ 5 suggests multicollinearity between variables [35]. In the female models, VIF ranged between 1.73 and 2.28, while in the male models, VIF ranged between 2.08 and 2.19. HIV treatment was not considered in the models as a few of the participants reported HIV treatment (12%).

## Results

### Characteristics of the study participants

The characteristics of participants are described in Table 1. Of the one thousand one hundred and forty-three (1143) participants, 796 (70%) were women and 347 (30%) were men. The median age of women and men was 51 (46–56) and 50 (45–55) years respectively, with no significant difference between the sexes. The prevalence of overweight and obesity was more than three times higher in women (76%) than in men (21%) (p < 0.001). Although unemployment was higher among women (66%) than men (54%), more women than men fell into the highest SES quintile (p = 0.004). More men than women used tobacco (p < 0.001) and alcohol (p < 0.001). Men reported more MVPA than women (p < 0.001). The sleep duration per night was longer in men than women (p = 0.005) The participants were all African ethnicity and predominantly from the Ba-Pedi tribe (89%).

**Table 1.** Demographics, socio-economic, behavioural and biological characteristics by gender.

	Women (n = 796) n (%)	Men (n = 347) n (%)	p-values
**Response/outcome variable**			
BMI (kg/m^2^)*	30.1 (25.2–35.8)	20.6 (18.9–24.3)	< 0.001
BMI categories			
Underweight	24 (3)	69 (20)	< 0.001
Normal weight	166 (21)	205 (59)	
Overweight	198 (25)	63 (18)	
Obese	408 (51)	10 (3)	
**Socio-demographics**			
Age (years)	51 (46–56)	50 (45–55)	0.233
40–44	154 (19)	80 (23)	0.533
45–49	186 (23)	75 (22)	
50–54	201 (25)	87 (25)	
55–60	255 (32)	105 (30)	
Marital status			
Never married or cohabited	179 (23)	98 (28)	0.094
Married/living with partner	418 (53)	174 (50)	
Divorced/Widowed	199 (25)	75 (22)	
Level of education			
No formal education	72 (9)	21 (6)	0.116
Primary	268 (34)	109 (31)	
Secondary	432 (54)	200 (58)	
Tertiary	24 (3)	17 (5)	
Employment Status			
Unemployed	523 (66)	188 (54)	< 0.001
Employed	273 (34)	159 (46)	
SES			
1^st^ quintile	82 (10)	63 (18)	0.004
2^nd^ quintile	179 (23)	83 (23)	
3^rd^ quintile	109 (14)	43 (12)	
4^th^ quintile	182 (23)	64 (18)	
5^th^ quintile	244 (31)	94 (27)	
Housing density (People/room)	1.0 (0.7–1.3)	1.0 (0.7–1.7)	< 0.001
**Behavioural**			
Diet			
Bread intake (servings/day) *	1.7 (0.9–3.0)	1.7 (0.6–3.0)	0.138
Fruit and vegetable intake (servings/day)*	1.3 (0.9–2.3)	1.3 (0.9–2.3)	0.707
Sugar sweetened beverages (servings/day)*	0.3 (0.3–0.6)	0.3 (0.3–0.7)	0.047
Smoking status			
Never	743 (93)	52 (15)	< 0.001
Current smoked	24 (3)	220 (63)	
Former smoker	29 (4)	75 (22)	
Use alcohol			
Never	551 (69)	52 (15)	< 0.001
Current non-problematic	44 (6)	75 (22)	
Current problematic	56 (7)	139 (40)	
Former Consumer	145 (18)	81 (23)	
Physical Activity and Sleep			
MVPA (minutes/week)*	300 (200–480)	360 (180–600)	< 0.001
MVPA Categories			
Inactive (< 150minutes/week)	80 (10)	47 (13)	0.084
Active (≥ 150minutes/week)	716 (90)	300 (87)	
Sleep (hours/night)*	9.0 (8.0–10.0)	9.6 (8.4–10.3)	< 0.001
**Biological**			
HIV status			
HIV negative	612 (78)	267 (79)	0.647
HIV positive	175 (22)	71 (21)	
Number of pregnancies	5.0 (3.0–6.0)	-	-
Menopausal status			-
Pre-menopausal	255 (32)	-	
Peri-menopausal	81 (10)	-	
Post-menopausal	377 (48)	-	
Not staged	83 (10)	-	

*Not normally distributed data expressed as median (IQR) and categorical data expressed as n (%), SES – Socioeconomic status, MVPA – Moderate to Vigorous Physical Activity, HIV – Human immunodeficiency virus; missing HIV = 18

### Factors associated with BMI in bivariate linear modelling

Table 2 shows the bivariate associations of BMI with sociodemographic, behavioural and biological factors. In both men and women, a higher SES and being married or living with partner were significantly associated with an increased BMI. Women with secondary education were more likely to have a higher BMI than women with no formal education (p = 0.045). Bread intake (p < 0.001) and sugar-sweetened beverages (p = 0.005), were positively associated with BMI in women only. Former use of tobacco in men (p = 0.004) and current use of tobacco in both sexes (p < 0.001) were associated with a lower BMI when compared to those who never used tobacco. In men, former or current use of alcohol is significantly associated with lower BMI compared to not using alcohol, while in women, current problematic use of alcohol is significantly associated with a lower BMI compared to not consuming alcohol. A longer sleep duration was associated with a lower BMI in both sexes (p ≤ 0.002). HIV infected men (p < 0.001) and women (p = 0.012) were more likely to have a lower BMI.

**Table 2.** Bivariate linear modeling (unstandardized beta coefficient) for the association of BMI with demographic, socio-economic, behavioural, and biological factors for women and men.

	Women (n = 796)		Men (n = 347)	
	Coefficient (95%CI)	p-values	Coefficient (95%CI)	p-values
**Socio-demographic factors**				
Age	−0.002 (−0.004, 0.002)	0.273	0.001 (−0.001, 0.004)	0.482
Marital Status				
Never married or cohabited	Ref		Ref	
Married/living with partner	0.06 (0.02, 0.11)	0.008	0.08 (0.04, 0.12)	< 0.001
Divorced/Widowed	0.02 (−0.03, 0.07)	0.483	−0.005 (−0.06, 0.04)	0.842
Level of education				
No formal education	Ref		Ref	
Primary	0.03 (−0.04, 0.09)	0.391	0.02 (−0.06, 0.09)	0.707
Secondary	0.07 (0.004, 0.013)	0.036	0.02 (−0.05, 0.10)	0.551
Tertiary	0.03 (−0.09, 0.15)	0.627	0.09 (−0.03, 0.19)	0.133
Employment Status				
Unemployed	Ref		Ref	
Employed	0.01 (−0.02, 0.05)	0.488	0.02 (−0.01, 0.06)	0.188
SES				
1^st^ quintile	Ref		Ref	
2^nd^ quintile	0.05 (−0.01, 0.12)	0.107	0.005 (−0.05, 0.06)	0.857
3^rd^ quintile	0.09 (0.02, 0.17)	0.010	0.06 (−0.003, 0.12)	0.063
4^th^ quintile	0.09 (0.03, 0.16)	0.005	0.07 (0.009, 0.13)	0.023
5^th^ quintile	0.15 (0.09, 0.21)	< 0.001	0.09 (0.04, 0.15)	< 0.001
Housing density (People/room)	−0.01 (−0.03, 0.02)	0.611	−0.007 (−0.03, 0.02)	0.660
**Behavioural factors**				
Diet				
Bread intake (Servings/day)	0.02 (0.01, 0.03)	< 0.001	0.007 (−0.003, 0.02)	0.188
Fruit/vegetable intake (servings/day)	0.01 (−0.001, 0.02)	0.132	−0.0003 (−0.008, 0.007)	0.930
Sugar sweetened beverages (servings/day)	0.03 (0.01, 0.06	0.005	0.02 (−0.005, 0.04)	0.131
Smoking status				
Never	Ref		Ref	
Current smoked	−0.19 (−0.29, −0.08	< 0.001	−0.14 (−0.19, −0.09)	< 0.001
Former smoker	−0.04 (−0.14, 0.05)	0.394	−0.06 (−0.12, −0.002)	0.044
Use alcohol				
Never	Ref		Ref	
Current non-problematic	−0.01 (−0.08, 0.07)	0.901	−0.12 (−0.19, −0.06)	< 0.001
Current problematic	−0.10 (−0.17, −0.03)	0.005	−0.13 (−0.19, −0.08)	< 0.001
Former Consumer	−0.01 (−0.06, 0.03)	0.561	−0.07 (−0.13, −0.02)	0.014
Physical Activity and Sleep				
MVPA (minutes/week)	0.00008 (−0.0007, 0.00009)	0.843	−0.00002 (0.00008, 0.00004)	0.446
MVPA Categories				
Inactive (< 150min/wk)	Ref		Ref	
Active≥ 150min/wk)	−0.03 (−0.09, 0.03)	0.257	−0.03 (−0.08, 0.03)	0.326
Sleep (hours/night)	−0.02 (−0.03, −0.01)	< 0.001	−0.02 (−0.03, −0.007)	0.002
**Biological factors**				
HIV status				
HIV negative	ref		Ref	
HIV positive	−0.13 (−0.17, −0.08)	< 0.001	−0.06 (−0.10, −0.01)	0.012
Number of pregnancies	0.005 (−0.002, 0.01)	0.170	-	-
Menopausal status				
Pre-menopause	Ref		-	-
Peri	−0.009 (−0.07, 0.06)	0.779	-	-
Post	−0.04 (−0.12, 0.04)	0.313	-	-
Not staged	−0.05 (−0.11, 0.01)	0.125	-	-

SES – Socioeconomic status, MVPA – Moderate to Vigorous, Physical Activity, HIV – Human immunodeficiency virus, min/wk – minutes/week

### Factors associated with BMI in hierarchical multivariate linear modelling

The hierarchical model results for women and men are presented in Tables 3 and 4, respectively. In Model 1, sociodemographic factors with p < 0.2 in bivariate were included. For women, a significant positive association of upper SES quintiles and BMI was found. For men, BMI was positively associated with being married or living with partner (p = 0.003) and the highest SES quintile (p = 0.003). The sociodemographic factors explained 3.2% and 7.6% of the variance in BMI in women and men, respectively.

**Table 3.** Sex-stratified hierarchical models showing demographic, socio-economic, behavioural and biological factors associated with log BMI for women.

	Model 1 Demographic and socioeconomic factors		Model 2 Demographic, socio-economic and behavioural factors		Model 3 Demographic, socio-economic, behavioural and biological factors	
	Coeff (95%CI)	p-value	Coeff (95%CI)	p-value	Coeff (95%CI)	p-value
**Age (years)**	−0.002 (−0.005, 0.001)	0.280	−0.002 (−0.005, 0.001)	0.267	−0.004 (−0.008, −0.0009)	0.012
**Marital Status**						
Never married or cohabited	Ref		Ref		Ref	
Married/living with partner	0.05 (−0.001, 0.09)	0.053	0.05 (0.005, 0.09)	0.029	0.03 (−0.02, 0.08)	0.220
Divorced/Widowed	0.02 (−0.03, 0.08)	0. 390	0.03 (−0.03, 0.08)	0.338	0.03 (−0.03, 0.08)	0.345
**Level of education**						
No formal education	Ref		Ref		Ref	
Primary	0.02 (−0.05, 0.09)	0.560	0.003 (−0.06, 0.07)	0.922	0.005 (−0.06, 0.07)	0.887
Secondary	0.04 (−0.03, 0.11)	0.256	0.010 (−0.05, 0.08)	0.698	0.008 (−0.06, 0.08)	0.821
Tertiary	−0.03 (−0.15, 0.9)	0.654	−0.07 (−0.19, 0.05)	0.258	−0.07 (−0.19, 0.05)	0.248
**SES**						
1^st^ quintile	Ref		Ref		Ref	
2^nd^ quintile	0.05 (−0.01, 0.12)	0.117	0.04 (−0.02, 0.10)	0.279	0.03 (−0.04, 0.09)	0.370
3^rd^ quintile	0.09 (0.01, 0.17)	0.016	0.07 (−0.004, 0.14)	0.064	0.07 (−0.004, 0.14)	0.064
4^th^ quintile	0.09 (0.02, 0.15)	0.012	0.07 (−0.001, 0.13)	0.054	0.07 (0.007, 0.14)	0.031
5^th^ quintile	0.14 (0.07, 0.21)	< 0.001	0.11 (0.04, 0.17)	0.002	0.10 (0.03, 0.17)	0.003
**Diet**						
Bread intake (Servings/day)			0.01 (0.0004;0.02)	0.041	0.007 (−0.004, 0.02)	0.228
Fruit/vegetable intake (servings/day)			0.00 (−0.005, 0.01)	0.435	0.007 (−0.002, 0.02)	0.118
Sugar sweetened beverages (servings/day)			0.02 (−0.0008, 0.05)	0.058	0.02 (−0.003, 0.04)	0.081
**Smoking status**						
Never			Ref		Ref	
Current smoked			−0.14 (−0.25, −0.03)	0.010	−0.15 (−0.26, −0.05)	0.005
Former smoker			−0.02 (−0.12, 0.07)	0.638	−0.02 (−012, 0.08)	0.674
**Use alcohol**						
Never			Ref		Ref	
Current non-problematic			0.01 (−0.06, 0.09)	0.713	0.04 (−0.05, 0.11)	0.411
Current problematic			−0.06 (−0.13, 0.01)	0.108	−0.05 (−0.13, 0.02)	0.151
Former Consumer			0.005 (−0.04, 0.05)	0.840	0.0 (−0.03, 0.06)	0.482
**Sleep (hours/night)**			−0.02 (−0.03, −0.003)	0.013	−0.02 (−0.03, −0.003)	0.015
**HIV Status**						
HIV negative					Ref	
HIV Positive					−0.13 (−0.17, −0.08)	< 0.001
**Number of pregnancies**					0.003 (−0.005, 0.01)	0.445
R^2^	0.044		0.081		0.116	
Adjusted R^2^	0.032		0.059		0.092	
F (p-value)	< 0.001		< 0.001		< 0.001	
**Mean VIF (min, max)**	2.28 (1.20,3.56)		1.75 (1.07, 3.71)		1.73 (1.07,3.87)	

SES – Socioeconomic status, HIV – Human immunodeficiency virus

**Table 4.** Sex-stratified hierarchical models showing demographic, socio-economic, behavioural and biological factors associated with log BMI for men.

	Model 1 Demographic and socioeconomic factors		Model 2 Demographic, socio-economic and behavioural factors		Model 3 Demographic, socio-economic, behavioural and biological factor	
	Coeff (95%CI)	p-value	Coeff (95%CI)	p-value	Coeff (95%CI)	p-value
Age (years)	0.001 (−0.002, 0.004)	0.445	0.0007 (−0.002, 0.004)	0.643	−0.0004 (−0.003, 0.003)	0.816
**Marital Status**						
Never married or cohabited	Ref		Ref		Ref	
Married/living with partner	0.06 (0.02, 0.11)	0.003	0.08 (0.04, 0.12)	< 0.001	0.09 (0.05, 0.13)	< 0.001
Divorced/Widowed	−0.01 (−0.07, 0.04)	0.623	0.02 (−0.03, 0.06)	0.503	0.04 (−0.02, 0.09)	0.135
**Level of education**						
No formal education	Ref		Ref		Ref	
Primary	−0.009 (−0.09, 0.07)	0.816	0.009 (−0.07, 0.09)	0.803	−0.01 (−0.09, 0.06)	0.743
Secondary	−0.006 (−0.08, 0.07)	0.881	0.005 (−0.07, 0.08)	0.901	−0.02 (−0.09, 0.05)	0.589
Tertiary	0.02 (−0.09, 0.13)	0.674	0.04 (−0.07, 0.15)	0.448	0.006 (−0.10, 0.11)	0.911
**Employment status**						
Unemployed	Ref		Ref		Ref	
Employed	0.02 (−0.01, 0.06)	0.217	0.02 (−0.02, 0.06)	0.265	0.02 (−0.02, 0.05)	0.365
**SES**						
1^st^ quintile	Ref		Ref		Ref	
2^nd^ quintile	0.004 (−0.05, 0.06)	0.877	−0.00008 (−0.05, 0.05)	0.998	−0.005 (−0.05, 0.04)	0.839
3^rd^ quintile	0.05 (−0.02, 0.11)	0.153	0.04 (−0.02, 0.11)	0.174	0.04 (−0.03, 0.09)	0.257
4^th^ quintile	0.06 (−0.003, 0.11)	0.062	0.04 (−0.02, 0.09)	0.226	0.03 (−0.2, 0.09)	0.274
5^th^ quintile	0.09 (0.03, 0.14	0.003	0.07 (0.01, 0.12)	0.017	0.07 (0.02, 0.13)	0.009
**Diet**						
Bread intake (Servings/day)			0.002 (−0.008, 0.01)	0.640	0.002 (−0.008, 0.01)	0.679
Sugar sweetened beverages (servings/day)			0.009 (−0.01, 0.03)	0.373	0.007 (−0.01, 0.03)	0.506
**Smoking status**						
Never			Ref		Ref	
Current smoked			−0.10 (−0.16, −0.04)	< 0.001	−0.09 (−0.16, −0.04)	< 0.001
Former smoker			−0.04 (−0.11, 0.02)	0.188	−0.04 (−0.01, 0.02)	0.216
**Use alcohol**						
Never			Ref		Ref	
Current non-problematic			−0.07 (−0.13, −0.006)	0.032	−0.08 (−0.14, −0.01)	0.016
Current problematic			−0.07 (−0.13, −0.003)	0.041	−0.07 (−0.14, −0.01)	0.023
Former Consumer			−0.05 (−0.12, 0.01)	0.113	−0.05 (−0.11, 0.02)	0.149
**Sleep (hours/night)**			−0.008 (−0.02, 0.003)	0.142	−0.007 (−0.01, 0.003)	0.189
**HIV Status**						
HIV negative					Ref	
HIV Positive					−0.08 (−0.12, −0.03)	< 0.001
R^2^	0.105		0.218		0.248	
Adjusted R^2^	0.076		0.172		0.200	
F (p-value)	< 0.001		< 0.001		< 0.001	
**Mean VIF (min, max)**	2.08 (1.05,4.59)		2.19 (1.10,4.89)		2.16 (1.10–4.93)	

SES – Socioeconomic status, HIV – Human immunodeficiency virus

In model 2, behavioural factors with p < 0.2 were added to Model 1. For women, the model explained 5.6% of the variance in BMI. Furthermore, BMI was positively associated with being married or living with partner (p = 0.029) and highest SES quintile (p = 0.002), while negatively associated with current smoking (p = 0.010) and sleep duration (p = 0.013). For men, the model explained 17.6% of the variance in BMI. In addition, BMI was positively associated with being married or living with partner (p < 0.001) and highest SES quintile (p = 0.017), while negatively associated with current smoking (p < 0.001) and alcohol use (p = 0.032).

In Model 3, biological factors were added to Model 2. For women BMI was inversely associated with current smoking (p = 0.005), HIV positive (p < 0.001), sleep duration (p = 0.015) and age (p = 0.012), while positively associated with upper SES quintile (p = 0.003). The model explained 9.2% of the variance in BMI. For men, BMI was negatively associated with current smoking (p < 0.001), being HIV positive (p < 0.001) and alcohol use (p = 0.016), but was positively associated with SES (p = 0.009) and being married (p < 0.001). The model explained 20% of the variance in BMI.

## Discussion

This study is novel for the DHDSS site in that, unlike previous reports [15,19,36], the relationship between BMI and a pool of associated factors, is reported. Obesity levels in this study are lower for both men and women when compared to an earlier study conducted in DHDSS [19]. Reasons for the difference between obesity levels are first, that the earlier study did not consider HIV infected people and second, age differences between to study samples. The major findings of this study are first, the marked difference in obesity levels between sexes, second a lack of association between PA and BMI and third, a positive association between SSB and BMI.

Women in our study are markedly more overweight-obese than men, a reflection of the trend in LMIC [8]. They are not inactive (as shown in this study and other previous objective studies in Dikgale) and early dietary surveys do not provide evidence of over-consumption, especially in women. However, overall carbohydrate (CHO) intake (which includes refined CHO and SSB) is nearly 70% and was shown to be higher in women than men [21]. A recent Prospective Urban and Rural Epidemiology (PURE) study has shown that poor health outcomes are significantly associated with a high CHO intake. Also, the level of stunting is high (48%) [22]. The high level of CHO intake could be linked to food insecurity in a low SES setting, thus greater reliance on cheap, refined foodstuffs (SSB, white bread, mealie-meal, sugar). A lack of definitive data on the influence of some of these factors on BMI in this community, suggests further investigation. The fluctuations in reproductive hormone concentrations throughout women’s lives uniquely predispose them to excess weight gain. Other factors such as alcohol use, tobacco use, that are shown to be more prevalent among men than women and are associated with decreased BMI may contribute to the observed difference.

An unexpected finding was the lack of association between PA and BMI in this study, despite a high proportion of participants being physically active. This finding concurs with recent reviews on the relationship of PA and obesity that suggested an absence of such a relationship [37,38]. Furthermore, work and domestic PA were not associated with obesity [39], supporting present findings. The commonly held position that increased physical activity is associated with a decrease in BMI [40,41] is in contrast with studies reporting positive associations between PA and BMI [15,16,42,43]. There is compelling evidence suggesting that PA has no influence of the risk of becoming overweight or obese [14], however, the value of PA on the general state of health is universally accepted [44,45].

Our study found that a shorter duration of sleep was associated with obesity in women but not men. Similarly, a study conducted in urban Soweto found the same association in women but not men [46]. Elsewhere, in Kenya and the USA, shorter sleep was associated with obesity in women only [47,48]. Evidence suggests that acute short sleep duration results in an imbalance of hormones controlling hunger and appetite, which in turn, are characterized by decreased leptin (satiety hormone), and increased ghrelin (appetite-stimulating hormone) [49,50].

Being a married man is associated with an increased likelihood of having a high BMI in Dikgale HDSS. Consistent with our findings, Nienaber-Rousseau et al. (2017) [51] found an association of married or cohabiting status and BMI in South African men but not in women. This finding is based on the hypothesis that having a partner is associated with eating more frequent and regularly [52,53] and that married men have greater regulation of health behaviour such as quitting smoking, changes that may increase BMI [54]. Interestingly, in a recent meta-analysis of two million participants, being a married man or woman was associated with a decreased risk of CVD when compared to being unmarried (never married, divorced or widowed).

This study found that a high SES was associated with an increased likelihood of being obese in both sexes. These findings are supported by a study from Soweto that found a direct association of SES with obesity among rural adult women, but found that among urban women the relationship was mediated by PI [16]. Another study from an urban African township in Cape Town, South Africa, showed that a high SES was associated with obesity in women but not in men [55]. Our findings are consistent with studies from Sub-Saharan Africa (SSA) [47,56]. In LMIC, there is evidence that people with low SES have limited resources, negatively affecting their food consumption [57]. Moreover, they are more likely to be involved in physically demanding jobs, while those with a higher SES may have sufficient resources for access to more food and may engage in less physically demanding occupations and may use mechanized transportation [57]. Alternatively, a low SES is associated with the consumption of low quality, highly processed and refined foods that are also cheap and affordable [58]. Interestingly, the relationship of SES and obesity in developed countries is reversed, with a high SES being associated with a lower BMI [55,57]. Unlike LMIC, a high SES status in developed countries is associated with a greater concern around the health costs of excess weight and associated diseases and concomitant healthier diets and other lifestyle interventions, which may not be accessible to a lower SES status [57].

Inverse relationships were observed between BMI and tobacco use, and HIV infection in both sexes. Our findings are congruent with other studies from South Africa [59] and SSA [47, 56]. Although tobacco use is associated with low BMI, the use of tobacco should be discouraged as it is detrimental to health through the increased risk of lung cancer and cardiovascular diseases [60]. In South Africa, evidence suggests a decline in the use of over the counter cigarettes probably due to increased taxation, but there is a shift towards the use of ‘roll your own’ cigarettes particularly in rural areas [61]. A lower BMI in treatment naïve HIV infected people, is likely due to disease associated cachexia [62], while a lower BMI in those on treatment may result from lipoatrophy caused by antiretroviral medication [63].

Alcohol use was associated with a decreased BMI in men in this study. Evidence on the relationship of alcohol use and BMI is conflicting, with studies reporting no associations [64], positive associations [65] and negative associations [66]. However, a recent review by Traversy and Chaput (2015) [67] suggests that variations in this relationship may be a result of differences in the types of alcohol consumed, amount of alcohol per occasion, frequency of drinking and the differences in the alcohol metabolism between age groups.

Although a previous study found SSBs among the food items appearing most frequently in the diet of Dikgale adults, our study is the first to assess the association of SSBs and BMI in this community. Sugar sweetened beverages (SSBs) tended to increase BMI in females but not in men. Kang and Kim (2017) [68] reported an association of SSBs with the Metabolic Syndrome only in women. The South African Adult PURE cohort study showed an association between higher consumption of added sugars and sucrose-sweetened beverages with increased non-communicable disease risk factors [69]. The role of SSBs in driving obesity and its associated complications has been supported in a recent review [70]. A lack of association between bread intake and BMI observed in this study is not congruent with other studies [47,71]. A previous study by Steyn (2001) [21] from Dikgale HDSS showed a high intake of carbohydrates such as, white bread, white sugar, maize porridge and white rice.

The strength of the study includes a relatively large sample size and the inclusion of variables not previously examined within the same analyses. Furthermore, this study provides baseline Dikgale HDSS cohort data for Phase 2 of the Africa Wits-INDEPTH Partnership for Genomic Study (AWI-Gen). However, some limitations need to be acknowledged. Thist study was cross-sectional in design which limits the ability to determine causal inference. Although we did not randomly sample the participants, we provided all 40–60 year permanent residents of Dikgale HDSS an equal opportunity to participate in the study by visiting their household and inviting them to participate. The potential recall bias from self-reported dietary and lifestyle variables and HIV status would likely have influenced the results. We acknowledge that our study does not include other important obesity-related modifiable and non-modifiable (genetic) risk factors. Future analyses should yield a more complete and nuanced understanding.

## Conclusion

Overweight and obesity was more prevalent in women than men. Our study detected no association between MVPA and BMI. Instead, a high SES was associated with high BMI, which might serve as a proxy to having sufficient resources for access to more food and sugar sweetened beverages (SSBs) that tended to increase BMI in females but not in men, and may be targets for educational programs to inform the general public of the harmful effects of high SSB intake. The current models explained for a 9.2% and 20% variance of BMI in women and men respectively. There is a need for further studies that explore the contribution of stunting, psycho-social factors, genetic factors, nutrition (detailed dietary surveys) to overweight and obesity in this community.

## Paper context

Rural DHDSS site is in northern Limpopo Province of South Africa. The high-obesity prevalence particularly in women, despite reports of high physical activity warranted further investigation of a broader coverage of obesity risk factors not previously assessed. The current findings point to a need to further explore the role of SSBs and detailed refined carbohydrate dietary patterns in addition to stunting, psycho-social factors, genetic factors, in the observed levels overweight and obesity in this community.
